# Supplementary material for: Tissue-specific expression of senescence biomarkers in spontaneously hypertensive rats: evidence of premature aging in hypertension
Source: PeerJ. 2023 Oct 19;11:e16300. doi: 10.7717/peerj.16300 (PMC10590574; doi:10.7717/peerj.16300)
Supplement: Supplemental Information 1 — The expression of senescence biomarkers in chronological aging in human and animal model in various organs and tissues. [file peerj-11-16300-s001.docx]

**Supplementary Table S1**

**Reference list for the expression of senescence biomarkers in chronological aging in human and animal models.**

| Organ/Tissue | Models | Senescence Markers | References |
| --- | --- | --- | --- |
| Heart | Mouse | - *p16^INK4a^,* and *p19^ARF^* increased with advanced age (up to 22 months). - Telomerase activity decreased in aged mice. | Torella *et al*., 2004 PMID: 14726476 |
|  | Mouse | - Telomere dysfunction increased with advanced age in cardiomyocytes (up to 30 months). - *p16^INK4a^* increased with aged mice (up to 24 months). | Anderson *et al*., 2019 PMID: 30737259 |
|  | Human | - p16^INK4a^ of cardiac progenitor cells (CPCs) was increased with advanced age. - Telomere length was reduced with age. | Lewis-McDougall *et al*., 2019 PMID: 30854802 |
| Liver | Mouse | - *Timp1*, *Mmp12*, *Cxcl1*, *Cxcl2*, *Ccl8*, *p16* and *p21* mRNA expression were upregulated with age in liver | Hudgins *et al*., 2018  PMID: 29527222 |
|  | Human | - p16^INK4a^ was significantly higher in aging populations (> 65 years old) | Zhu *et al*., 2014 PMID: 24325248 |
| Skeleton | Mouse and human | - p16^INK4a^ mRNA expression was upregulated in the chondrocytes of aging mice (up to 27 months) and human populations. | Diekman *et al*., 2018  PMID: 29744983 |
|  | Mouse and human | - p16^INK4a^ mRNA expression was significantly higher with aging in osteoblasts and osteocytes of mice. - p21 mRNA expression was upregulated in osteocytes of aging mice (up to 24 months). - IL-6 mRNA expression was upregulated in osteocytes of aging mice. - p16^INK4a^ and p21 mRNA expression was upregulated in aging human (76 years old) | Farr *et al*., 2016 PMID: 27341653 |
| Kidney | Mouse | - *Il1a*, *Mmp3*, *Mmp12*, *Cxcl1*, *Cxcl2*, and *p21* mRNA expression were upregulated with age in kidney | Hudgins *et al*., 2018  PMID: 29527222 |
|  | Human | - p16^INK4a^ mRNA and protein expression were upregulated with kidneys of aging populations. - p16^INK4a^ expression was correlated with age. | Melk *et al*., 2004  PMID: 14717921 |
| Brain | Mouse | - A list of 20 cytokines was found to be upregulated in ventral hippocampus. | Porcher *et al*., 2021 PMID: 34551810 |
|  | Mouse | - *Mmp12* and *Timp1* expression were upregulated with age in the right cerebral hemisphere of aged mice (up to 18 months). | Lui *et al*., 2013 PMID: 23159549 |
|  | Mouse | - *Il1a*,*Timp1*, *Mmp12*, *Cxcl1*, *Cxcl2* were upregulated with age in hypothalamus | Hudgins *et al*., 2018  PMID: 29527222 |
| Blood | Human | - Serum IL-6 was increased with age. | Wei *et al*., 1992  PMID: 1453878 |
|  | Mouse/Rat/Human | - Serum IL-6 and IL-6 receptors were increased with age. | Ershler and Keller, 2000 PMID: 10774463 |
|  | Human | - Serum IL-6 was positively correlated with age in males. | Young *et al*., 1999 PMID: 10469050 |
|  | Human | - Serum Timp1 markedly increased after 65 years old. | Ishikawa *et al*., 2019 PMID: 33693092 |
| Others | Mouse | - *p16^INK4a^* expression in cecum, ovary, and uterus was increased with age (up to 26 months). | Krishnamurthy *et. al*., 2004  PMID: 15520862 |
